# Internal Structure of Dietary Habits as a Restriction on Healthy Eating Policy in Japan

**DOI:** 10.3390/nu16142296

**Published:** 2024-07-17

**Authors:** Makoto Hazama, Kouji Satoh, Mari Maeda-Yamamoto, Jun Nishihira

**Affiliations:** 1 Department of Medical Management and Informatics, Hokkaido Information University, Ebetsu 069-8585, Japan; m-hazama@do-johodai.ac.jp (M.H.);; 2 Institute of Food Research, National Agriculture and Food Research Organization, Tsukuba 305-8642, Japan

**Keywords:** food consumption pattern, internal structure of dietary habit, healthy eating policy, statistical model

## Abstract

Although promoting healthy eating is a policy objective, the manageability of dietary habits remains uncertain. Personal dietary patterns reflect many factors, some of which are relatively manageable for individuals whilst others are not. In this article, assuming that some sort of information about the manageability of dietary habits is contained in the observed patterns of food consumption, we focused on dietary patterns on their own. We introduced a statistical descriptive model for data from a food frequency questionnaire, estimated the strength of pairwise linkage between foodstuffs, and grouped foodstuffs by applying community detection to the networks of the estimated inter-food linkages. Those linkages represent the co-movement of pairs of food in consumption. Furthermore, we demonstrated an analysis of the relationship between mental health and dietary habits, considering the aspect of the manageability of dietary habits. Using an observational study in Japan, we obtained the following results: 115 foodstuffs were divided into three groups for both genders, but the compositions were different by gender; in the analysis of mental and physical health, some stress response items were associated with a dependence on some of those food groupings (e.g., “extremely tired” was negatively associated with a group containing tomatoes, cucumber, mandarin, etc., for female subjects). As the grouping of foodstuffs based on our estimation depicted an internal structure of dietary habit that a healthy eating policy could regard as a constraint, it follows that we should design such a policy along the same lines as that grouping.

## 1. Introduction

In the general context of investigating the relationships between dietary habits and health, motivated by healthy eating policies, it is assumed that dietary habits are a manageable means of achieving a healthy life. But what sense do “manageable dietary habits” have? As pointed out by researchers, food choices are affected and complicated by a variety of factors [1]. Among these factors of food choice, some are external or exogenous and others are internal or endogenous, depending on the standpoint of decision makers. For instance, sociocultural factors such as economic variables are external, and cognitive factors such as attitudes, likes, and preferences are internal for individual persons, while these are the opposite for policy makers. In some cases, in the real world, decisions regarding food choice are “seen as heavily influenced by factors outside the control of the individual” [2]. Therefore, any control over dietary habits with the aim of a healthy life might be, to a greater or lesser extent, restricted by several factors influencing food choices.

From the perspective of modifying dietary habits within the context of healthcare, we particularly focused on the internal structure of dietary habits as represented by food consumption patterns. A restriction on the manageability of dietary habits would be, conceptually, reflected as a range of probable choices in a food choice space. If someone arbitrarily tries to change their dietary habits with free movement in a food choice space, ignoring their conventional food choice range, that practice would be burdensome for them and not a long-lasting diet modification.

In an improvement of diet, for example, if a person is advised to eat more tomatoes and she makes an effort to do so, then she is likely not only to increase the amount of tomatoes she intakes but also to change her intake of other foods. It might be because she likes to eat some foods with tomatoes and dislikes eating other foods with tomatoes, or because her lifestyle or custom imposes her to do so. In other words, peoples’ dietary habits are formed by their tastes, preferences, traditions, or other factors. The same presumably holds true in terms of nutrients (e.g., lycopene) instead of food (e.g., tomatoes). This implies that modifying dietary habits should consider the possibility of an external change in the intake of one food provoking unintended internal changes in the intake of other foods, which is induced by the internal structure of dietary habits caused by many factors. As those factors influencing food choices are not only explicitly but also implicitly imbedded in decision making [3], focusing on food consumption patterns alone as a representation of the internal structure of dietary habits enables a simple approach.

With the perspective discussed above, this article investigates the relationship between dietary habits and mental health based on an observational study in Ebetsu city, Japan. This study includes items from outcome scales of occupational stress measures, a food frequency questionnaire (FFQ), and physical and demographic attributes, among others, for each participant. In the focused relationship, which was captured by the regression of stress degrees on dietary habits and other covariates, independent variables for dietary habits were devised to deal with the internal structure problem. In particular, we introduced a statistical descriptive model, assuming that in the FFQ data, observations for each pair of foods are generated by a bivariate multinomial distribution. This allows us to estimate the degrees of the simultaneous intake of two foods by gender. Then, we divided foods into some groups according to the propensity of simultaneous intake. Finally, ordered logit regressions of the occupational stress measures on degrees of dependence on each group of foods were conducted, controlling for confounders. This setup is intended to provide an example of group-level insight into the relationship between food intake and health, rather than focusing on individual food items. This approach might enable dietary habit management to be more careful, meticulous, and, hence, long-lasting for practitioners.

According to our results, males and females showed distinctive characteristics in their dietary patterns. Some foodstuffs were intensively combined with other foodstuffs and played a key role in the subjects’ dietary habits. Based on the pairwise connectivity between foodstuffs, internal structures of dietary habit manifested as three groups of foods for both genders; in addition, the contents of each group were different by gender. Dependencies on some food groupings were shown to be associated with some aspects of mental health after adjusting for confounders. This demonstration provided a way to find the target group of foodstuffs that internally co-move together in a healthy eating policy.

## 2. Materials and Methods

### 2.1. Participants and Study Design

We exploited secondary data derived from ‘the comprehensive survey to establish an integrated database of food, gut microbiome, and health information (*Sukoyaka* Health Survey)’. A detailed explanation of the study protocol has been previously reported [4]. Although the *Sukoyaka* Health Survey was implemented in five municipalities in Hokkaido, Tokyo, Kyoto, Nagasaki, and Miyazaki prefectures across Japan, we only used a subsample of Hokkaido, focusing on one region and relying on the predominant integrity of that subsample. Data collections occurred in summer 2019 and winter 2020.

The derived dataset includes cross-sectional data on men and women in their 20s to 70s located in Ebetsu city, Hokkaido, Japan. The exclusion criteria were persons with serious cerebrovascular, heart, liver, kidney, or gastrointestinal diseases, infections requiring notification, blood donation within a certain period (within 16 weeks for women donating 400 mL, the last 12 weeks for men donating 400 mL, the last 4 weeks for 200 mL donation, and the last 2 weeks for component donation), and pregnant or breastfeeding women.

From 803 participants including 211 males and 592 females in the subsample of Hokkaido, we excluded 1 male and 2 females because of missing data form the FFQ, so we were left with 210 males and 592 females in the dataset. This is the sample that we used for the first part of our analysis. When we conducted regression analysis as the last part of our analysis described below, the sample size declined to 205 males and 587 females with completed data of the dependent and independent variables in the regression. Regarding the latter sample, mean age of female subjects is 50.1 (S.D. 11.3), while that of male subjects is 53.4 (S.D. 12.6).

This study was approved by the Ethics Committee of the Hokkaido Information University (approval date: 22 February 2024; approval number: 2023-11), and written consent was obtained from the participants. The research was conducted in accordance with the Helsinki Declaration.

### 2.2. Data Analysis

#### 2.2.1. Statistical Model

We employed a statistical descriptive model to deal with the internal structure of dietary habits in food–health analysis. This model is suitable for our stratified data for food intake frequency, which prevent us to employ a more simple and convenient analysis, such as principal component analysis.

For a generic pair of two foodstuffs, i and j, on every eating occasion, the intake of these foods was assumed to be determined by the four-states Bernoulli trial: food i without food j is eaten with probability $p_{1}$, food j without food i is eaten with probability $p_{2}$, both food i and j are eaten with probability $p_{3}$, and both of them are not eaten with $1-p_{1}-p_{2}-p_{3}$. The pair of frequencies of food i and j intakes during a given period follow bivariate multinomial distribution with parameters $p=\left(p_{1},\;p_{2},\;p_{3}\right)$, and the number of trials is $n_{0}$. In particular, the number of cycles of the Bernoulli trial is set at $n_{0}=21$, corresponding to weekly frequency of food intake (3 times a day for 7 days). Since our data include observations with higher frequency strata than $n_{0}$ times intake a week (Table A1 in Appendix A), we introduced into the model an irregular mode into which the food i intake switches independently with probability $q_{1}$. $q_{2}$ denotes the probability of food j’s higher-frequency mode. If food i is in higher-frequency mode and food j is not, the other food (j)’s intake occurs with probability $p_{3}$, conditional on both food’s mode (unconditional probability is $p_{3}q_{1}\left(1-q_{2}\right)$). See Appendix B for the log likelihood function.

Straightforwardly, the estimated values $\hat{p_{3}}\times n_{0}$ for each pair of foods provide an indicator of closeness between the two foods (the circumflex ^ means the estimated value of the parameter). On average, these two foods are simultaneously consumed $n_{0}\hat{p_{3}}$ times a week. However, for each pair of two foods, if either of the foods are more frequently consumed, the chance of two foods meeting on the same dining table is larger. Two foods on the same table may be independently picked out or may be chosen collectively. In our model, we could distinguish between the preferred/intended and the incidental/unintended combination of two foods. To distinguish the two possibilities, we test a null hypothesis that the estimated model has generated lesser simultaneous intakes of two foods than the special case model in which two food intakes are independent. In the special case model, assuming that foods i and j are consumed independently at every trial with probability $\pi_{1}$ and $\pi_{2}$, respectively, the incidental combination of them occurs with probability $\pi_{1}\pi_{2}$ (Appendix C). As the special case of the estimated model, the relations of $\pi_{1}=p_{1}+p_{3}$ and $\pi_{2}=p_{2}+p_{3}$ hold, so the null hypothesis is $H_{0}:\;p_{3}\leq \left(p_{1}+p_{3}\right)\left(p_{2}+p_{3}\right)$. We calculated the *p*-value of this non-linear hypothesis by simulation: we drew 1000 samplings of $\hat{p}$ from $N\left(\hat{p},\;\hat{\mathrm{Var}\left(\hat{p}\right)}\right)$ and evaluated the null ($N\left(\mu ,\;V\right)$ denotes the multivariate normal distribution with mean μ and covariance matirix V). For significant pairs of foods with level α=0.01, we assigned each of these pairs with the value $\delta \equiv \left(\hat{p_{3}}-\hat{\pi_{1}}\hat{\pi_{2}}\right)\times n_{0}$ as a measure of closeness between two foods in the internal structure of dietary habits. δ is nothing less than “excess degree of combination” of the food pair.

#### 2.2.2. Analysis

We conducted the estimation and testing of the two-food models for subsamples divided by gender. In FFQ data, participants were asked about their frequency of intake for 128 foods in the same strata choice format. The strata are shown in the second column of Table A1. Among the 128 food items, 17 items are duplicates in terms of foodstuff and are distinct in recipes. We consolidated these 17 items into 4 items (beef, pork, chicken, and bean curd) in line with a rule described in Appendix D. Then, we have 115 foods in our analysis (listed in Table A2). Unfortunately, these 115 foods do not include rice, miso soup, and drinks including alcohol, since these items are asked in different strata choice format. We were compelled to exclude the principal food for Japanese people as well as beverages from our analysis.

Although our model would generate data of an exact number of times of food intake, the FFQ in our study is stratified data. Coping with this, we specify the range of frequency for each stratum and calculate likelihood functions for stratified data by summing likelihoods over the interval for each stratum. The specification of the class interval is shown in Table A1.

With the results of the estimated model discussed earlier, we constructed networks of foods in which a vertex denotes a food item and an edge denotes the average degree of combination $\hat{p_{3}}\times n_{0}$ of two food items. This network includes only the edges with 1%-significant positive δ. Then, we classified the foods according to the result of community detection in the network [5]. Since the network community analysis maximizes the modularity, which takes into account connectedness even under randomness, we use $\hat{p_{3}}\times n_{0}$ instead of δ for the edge weights.

Food items in the same community are seen as closely related in combinatorial intake, and foods in different communities are more likely separately consumed. The co-movement of foods within a community and the separation of foods across communities make sense of the manageability of dietary habits. Groupwise alteration of eating would be more stress-free than individual food modification.

Therefore, eventually, in our analysis, we demonstrated an application of food–health analysis considering the internal structure of dietary habits. In particular, the association of mental health with dietary habits was estimated by an ordered logit model adjusted for age, housemates, occupation, etc. In this analysis, mental health is measured by responses to question items from the category of stress response in the occupational stress test “the Brief Job Stress Questionnaire” [6,7]. The explanatory variables of dietary habits are the dependence of energy, carbohydrates, protein, and lipids on each group of foodstuffs divided in the foregoing analysis. Since FFQ data capture both the frequency of food intake and the portion size consumed during each eating occasion, we can calculate energy and nutrient intake for individual food items. The four types of dependency measures based on energy, carbohydrates, protein, and lipids are exploited for a robustness check. Note that the denominator of the dependency variables is the total intake of energy, carbohydrates, protein, or lipids, including other food items (rice, miso soup, beverages), which were excluded from the 115 food items in our model estimation. We could use the information of the total intake of energy, etc., included in the secondary data of the *Sukoyaka* Health Survey.

By looking at the relationship between health and food groups based on their combinatorial consumption, we can consider the implication of dietary habits for health as being conditional on the restriction of the propensity of simultaneous intake imposed on the dietary habits.

## 3. Results

### 3.1. Model Estimation

For each of the 6555 pairs of two food items out of 115 foodstuffs, and for each subsample by gender, we estimated the bivariate multinomial distribution model discussed above. Table 1 shows the number of pairs in which the excess degree of combination δ is significantly greater than 0. For each significance level of α = 0.01, 0.05, and 0.10, the number of significant pairs for female subjects is nearly twice as many as for male subjects.

The top 10 pairs for 1%-significant values of δ are shown in Table 2. For female subjects, on average, out of $n_{0}$ = 21 meals in a week, carrots and onions are simultaneously consumed $n_{0}\hat{p_{3}}$ = 1.77 times, only carrots are consumed $n_{0}\hat{p_{1}}$ = 0.98 times, and only onion is consumed $n_{0}\hat{p_{2}}$ = 1.94 times. As discussed in the previous section, the simultaneous consumption 1.77 times might include both intended and unintended choices. At least, however, δ = 1.28 times out of 1.77 can be seen as intended simultaneous consumption. Combinations of onion with carrots, cabbage, tomatoes, long green onions, or radishes precede for male and female.

### 3.2. Community Analysis

**Figure 1:**
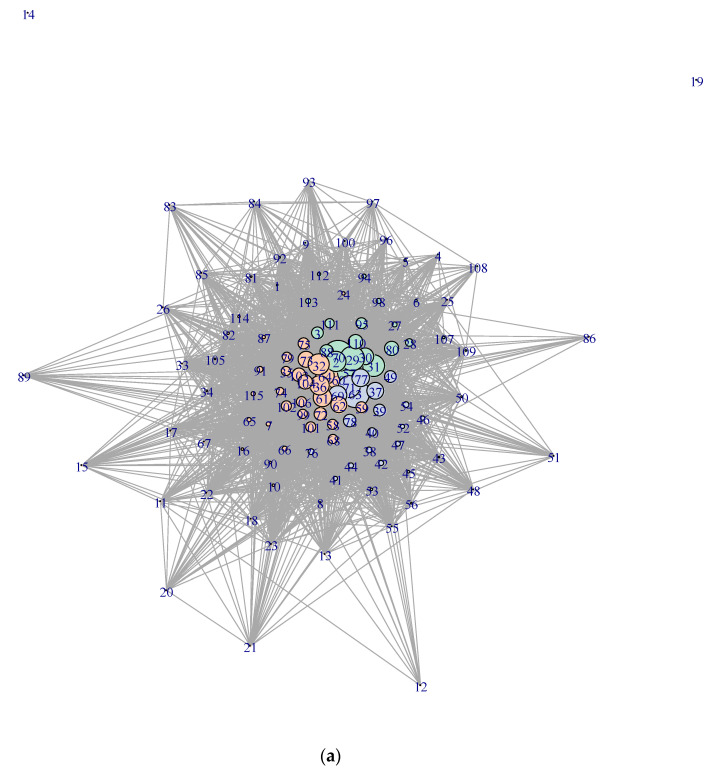

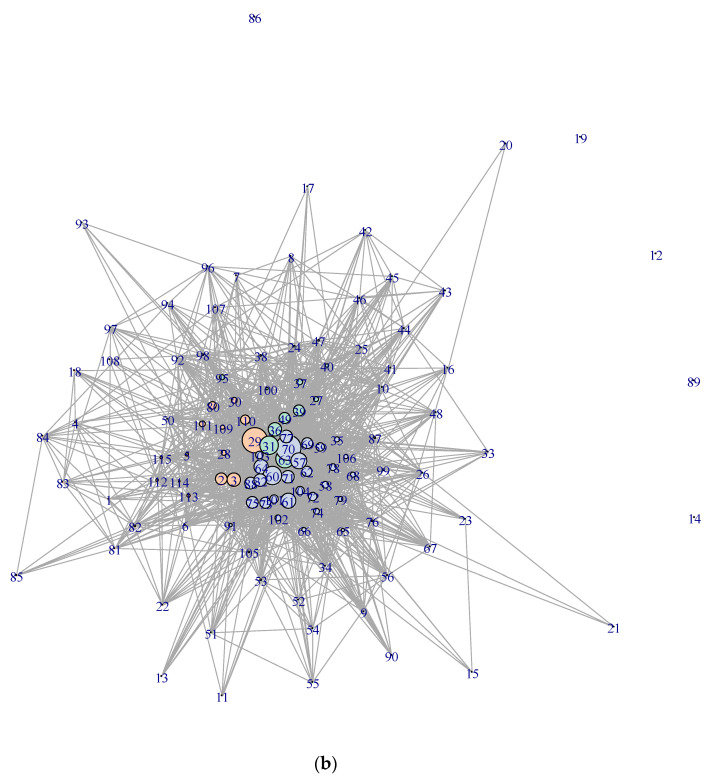
(**a**) Food combination network for female subjects. See Table A2 for label numbers of nodes. The size of each vertex is proportional to the sum of the degree of simultaneous intakes $\sum \hat{p_{3}}n_{0}$. (**b**) Food combination network for male subjects. See Table A2 for label numbers of nodes. The color of each vertex indicates the grouping of foods. The size of each vertex is proportional to the sum of the degree of simultaneous intakes $\sum \hat{p_{3}}n_{0}$.

The results of community analysis in the food combination networks are presented in Table 3 and Figure 1. According to Table 3, groupings of foods exhibit both affinity and differences between male and female subjects. For instance, carrot and onion are in the same group for male and female subjects in common; egg and onion are in the same group for female subjects but not for male subjects; females combine tomatoes and cucumber, but males do not; and so on. In addition, sea breams and eel are isolated for male and female subjects. For male subjects, amberjack, rice cakes, and taro are also not combined closely with other food items.

All vegetables except tomatoes are in the same group for males (group 3 of males), while vegetables are divided across groups for females. All fishes except canned tuna are in the same group for females (group 2 of females), but fishes are scattered across several groups for males. All fruits and pickles belong to the same group for both genders, except pickled turnip for male subjects.

Figure 1 makes up for the inadequacies of the impression from Table 3. Although the detection of community structure in networks provides clear-cut grouping, the intra-community closeness among food items is not uniform. For each group of food items denoted by vertex color, foodstuffs are separated into central ones and peripheral ones. The former foods have many links with others and organize the community. The latter foods have links mainly with the former and so are accessional in the network community. The central foodstuffs in the entire networks are onion, tomatoes, egg, carrot, etc., for female subjects, and onion, egg, yogurt, tomatoes, etc., for male subjects.

A closer look at the data behind the network plots reveals the distribution of intra- and inter-community linkages. Foodstuffs with higher intra-community centrality are onion, egg, cabbage, bean curd, etc., for female subjects, and tomatoes, cucumber, lettuce, broccoli, etc., for male subjects. Foodstuffs with higher inter-community centrality are tomatoes, onion, cucumber, carrot, etc., for female subjects, and egg, onion, cabbage, yogurt, etc., for male subjects. See the Supplementary Table S1 for detail.

If we summarize the grouping by typical elements of foodstuffs, the groupings for each gender are as follows. For female subjects, group 1 consists of onion, egg, yogurt, pork, etc.; group 2 consists of cabbage, bean curd, radish, fermented soybeans, etc.; group 3 consists of tomatoes, cucumber, mandarin, lettuce, etc. For male subjects, group 1 consists of tomatoes, apple, banana, yogurt, etc.; group 2 consists of egg, salad dressings, bread, chicken, etc.; group 3 consists of cucumber, lettuce, broccoli, onion, etc. Since these food items play a central role within their respective groups, consciously altering the consumption of these foods is likely to lead to unintentional changes in the intake of other foods within the same group.

### 3.3. Ordered Logit Regression

The results of ordered logit regression are summarized in Table 4. The summary statistics of samples for ordered logit analysis are provided in Supplementary Table S2. In Table 4, the rows show 29 question items from the category of stress response in the occupational stress questionnaire [6,7]. These question items are measured by a four-point Likert scale (0 = “Almost never”, 1 = “Sometimes”, 2 = “Often”, and 3 = “Almost always”). Note that, depending on question items, the value of each answer should be interpreted inversely as a stress measure. Though the ordered logit model should be evaluated in terms of marginal effects on probabilities, we saw the relationship between mental health and dietary habits by the signs and significances of the coefficients of ordered logit regression for the sake of simplicity.

According to Table 4, the robust results are as follows: the item “I have felt extremely tired” is negatively associated with food group 3 (tomatoes, cucumber, mandarin, lettuce, etc.) for female subjects; the item “I have felt worried or insecure” and “I have felt gloomy” are positively associated with food group 1 (onion, egg, yogurt, pork, etc.) for female subjects; the item “I have felt restless” is positively associated with food group 2 (cabbage, bean curd, radish, fermented soybeans, etc.) for female subjects; the items “I have felt angry” and “I have felt tense” are negatively associated with food group 3 (cucumber, lettuce, broccoli, onion, etc.) for male subject; the items “I have experienced stomach and/or intestine problems” and “I have experienced diarrhea and/or constipation” are negatively associated with food group 1 (tomatoes, apple, banana, yogurt, etc.) for male subjects; all of these are with adjusting for confounders.

Regarding confounders in the regression analysis, we obtained the following robust results: conditional on other confounders, age is negatively associated with almost all items in scales of anger/irritability, fatigue, and anxiety for male and female subjects and depression only for male subjects; BMI is positively associated with several items in scales of anger/irritability and physical stress reaction for female subjects; caregiving is positively associated with several items in scales of anger/irritability, fatigue, anxiety, and depression for female subjects and fatigue, anxiety, depression, and physical stress reactions for male subjects (Supplementary Table S3).

## 4. Discussion

We estimated dietary patterns for each gender based on a statistical model. The patterns of combinations of foodstuffs are different between male and female subjects. Females showed greater persistence in combining food items than males (Table 1). The fact that females traditionally cook more often than males might be behind this result.

The pairwise linkage of food items expressing the propensity of combinatorial/simultaneous intake of two foodstuffs presented the internal structure of dietary habits. According to this structure, we grouped food items by network community detection analysis. Some foodstuffs were isolated in the networks: sea breams and eel for both genders. Traditionally, these foods are associated with festive occasions or particular seasons, and are therefore rare in Japan.

Our method with a model of the simultaneous intake of two foodstuffs and network community detection can be seen as a device of data dimension distraction. If we had numerical data on food intake frequency rather than stratified data as in the FFQ, we could perform principal component analysis. It would be expected that several components reflect relatively manageable variations in dietary habits and that the others reflect relatively unmanageable ones. At the least, manageable and unmanageable variations in food intakes are likely orthogonal to each other. In our framework with stratified data, we segregated several sets of axes along which some groups of food items co-move. For example, onion, egg, and yogurt co-move together for female subjects; cabbage, bean curd, and radish co-move for female subjects; tomatoes, apple, and banana co-move for male subjects; egg, salad dressings, and bread co-move for male subjects; etc. (Supplementary Table S1). Thus, the food groupings derived from our analysis are seen as dimensions along which changes in dietary habits are less burdensome for food combination habits. Therefore, in the framework of health as a dependent variable and dietary habits as independent variables, it is straightforward to use these measures by group for dietary habits, which was the reason behind our ordered logit regression of mental health on diet.

As for the results of the regression analysis (Table 4), for male subjects, the food group including all fruit items and the food group including almost all vegetable items were negatively associated with several stress reactions, which is comparable to the positive association of fruits and vegetables with well-being [8]. While it is pointed out that intake of fishes is positively associated with vigor for working people [9], the food group including almost all fishes was not negatively correlated with stress items for females in our sample.

On a theoretical basis, an additional remark on our methodology is appropriate. The parameter of our model δ is closely related to economic concepts of complementarity and substitutability between two foodstuffs. Despite the fact that the definition of the latter is built in terms of cross-price elasticity within demand system of foods, it seems possible that positive significant δ corresponds to complementarity, and negative significant δ, though we did not focus on this in our analysis, corresponds to substitutability. Actually, in the various disciplines related to food consumptions, estimations of complementarity and substitutability among foods have been conducted applying the method of the Almost Ideal Demand System [10,11] and others [12].

The difference between the two concepts is merely about what they represent: δ is about eating, and the two economics concepts are about purchasing. However, the correspondence between δ and complementarity/substitutability might be dissolved by the preservative quality of some foods. If consumers could buy some preservative foods at the time of their low price regardless of prices of related foods, the complementarity or substitutability might be underrepresented. Our method has an advantage of focusing directly on consumption rather than purchase.

This article has several limitations. First, in our analysis of the internal structure of dietary habits, some important food items, i.e., rice, miso soup, and beverages (alcohol, teas, coffee, fruit/vegetable juices, etc.), were excluded because of inconsistencies in the data format. If rice was able to be included in Figure 1, rice must have had greater centrality in the network, i.e., have large frequency of combinatorial intake with many other foods. Although the result of community detection in the food network (Table 3 and Figure 1) is critically affected by the exclusion of those food items, the result of each pairwise estimation of the simultaneous intake model (Table 1 and Table 2) is unaffected by the exclusion. Individual results on the co-movement of food pairings are still valid.

Second, the acceptability of the results of model estimation are mixed. The results in Table 2 contained some estimated values that are difficult to interpret. For example, pork and chicken for male subjects have values of $n_{0}\hat{p_{3}}$ = 1.65 and δ = 1.25 (*p*-value = 0.00), literally meaning that pork and chicken are simultaneously eaten 1.65 times a week, broken down into at least 1.25 incidents of intentional combinatorial intake and, at most, 0.40 incidents of incidental simultaneous intake. This relatively high combination of pork and chicken hardly fits the actual societal experience in Japan. It might be a spurious correlation, or, so far as meat and bean curd are concerned, might be alleviated by an adjustment of the method of data consolidation (Appendix D). As an extension, the model can be estimated using Bayesian methods with a prior distribution that incorporates external information about reasonable combinations of foods in general Japanese recipes.

Third, we estimated our model on a weekly basis, i.e., $n_{0}=21$ occurrences of meal occasions in 7 days, although we could have chosen a monthly basis. This decision was driven by the significant time consumption of computer processing. Saving time costed some loss of information precision. In particular, the strata of “less than 1 per month” and “1~2 per month” were consolidated to “0 per week” by our choice (see Table A1).

Forth, in the analysis of the internal structure of eating, we only conditioned gender by dividing the sample based on gender, despite the availability of demographic variables such as age, education, occupational status, etc., in our survey. These personal-state factors are important for dietary habits. To keep the subsample size appropriate, however, we avoided enrichment of subsample conditioning.

Fifth, because our survey was enhanced by applicants’ cooperation, our sample resulted in an imbalanced gender composition. That fact limits the comparison between male and female subjects in our analysis.

## 5. Conclusions

Our study proposes a novel method for extracting information about the internal structure of dietary habits from FFQ data. When considering dietary habits as a measure of public health policy, this internal structure becomes a constraint for managing food intake. To make the practical implementation of food choice improvement more palatable and less burdensome for practitioners, it is essential to take into account the internal structure of diet as a constraint in healthy eating policies.

## Figures and Tables

**Table 1:** The number of significant combinations.

α	0.01		0.05		0.10	
Female	3560	(54.3%)	4417	(67.4%)	4863	(74.2%)
Male	1716	(26.2%)	2527	(38.6%)	3110	(47.4%)

Note: The number of pairs with a significant positive excess degree of combination δ by significant level α. The number of all pairs of two foods out of 115 items is 6555.

**Table 2:** Top 10 list of excess degree of combination δ.

**Female**								
** Food i **	** Food j **	$n_{0}\hat{p_{1}}$	$n_{0}\hat{p_{2}}$	$n_{0}\hat{p_{3}}$	δ	***p*-Value**	$\hat{q_{1}}$	$\hat{q_{2}}$
carrot	onion	0.98	1.94	1.77	1.28	0.00	0.01	0.01
tomatoes	cucumber	2.23	0.96	1.64	1.16	0.00	0.02	0.00
pork	chicken	1.71	1.33	1.42	1.01	0.00	0.00	0.00
long green onion	onion	1.02	2.30	1.42	0.99	0.00	0.00	0.01
cabbage	onion	1.15	2.26	1.44	0.98	0.00	0.01	0.01
tomatoes	onion	2.31	2.13	1.59	0.90	0.00	0.02	0.01
cabbage	radish	1.47	0.75	1.11	0.88	0.00	0.01	0.00
tomatoes	lettuce	2.63	0.90	1.24	0.85	0.00	0.02	0.00
radish	onion	0.70	2.53	1.16	0.83	0.00	0.00	0.01
carrot	cabbage	1.57	1.41	1.17	0.83	0.00	0.01	0.01
**Male**								
** Food i **	** Food j **	$n_{0}\hat{p_{1}}$	$n_{0}\hat{p_{2}}$	$n_{0}\hat{p_{3}}$	δ	***p*-Value**	$\hat{q_{1}}$	$\hat{q_{2}}$
pork	chicken	1.27	1.21	1.65	1.25	0.00	0.00	0.00
carrot	onion	0.65	1.69	1.58	1.23	0.00	0.00	0.01
long green onion	onion	0.70	1.77	1.52	1.17	0.00	0.00	0.01
egg	onion	2.89	1.44	1.88	1.12	0.00	0.03	0.01
tomatoes	onion	1.56	1.80	1.44	0.98	0.00	0.00	0.01
cabbage	onion	1.39	1.91	1.35	0.93	0.00	0.00	0.01
egg	cabbage	3.21	1.24	1.55	0.92	0.00	0.03	0.00
carrot	radish	1.18	0.67	1.04	0.86	0.00	0.00	0.00
radish	onion	0.59	2.12	1.12	0.86	0.00	0.00	0.01
egg	pork	3.22	1.45	1.52	0.85	0.00	0.03	0.00

**Table 3:** Food groups divided based on propensity for combination.

	1	2	3	4	5
1	yogurt (29.0, 33.2), chocolate (8.2, 17.7), low-fat milk (8.5, 8.5), rice cracker (3.7, 8.2), biscuit, cookie (1.7, 7.5), peanuts (5.5, 2.6), Japanese cake (1.9, 3.4), ice cream (1.4, 3.2), snacks (1.6, 2.9), cakes (0.3, 1.3)	fermented soybeans (20.5, 30.0), fish sausage (chikuwa) (3.4, 5.9), salmon, trout (1.8, 4.4), pacific saury, mackerel (0.9, 4.8), boiled fish paste (kamaboko) (1.9, 2.1), fried fish paste (satsuma-age) (1.7, 1.4), clam, corb shell (0.7, 1.9), shirasuboshi (0.3, 2.0), squid (0.1, 0.6), octopus (0.1, 0.5)	tomatoes (28.9, 44.2), mandarin (10.5, 25.9), apple (17.1, 19.2), banana (17.6, 18.1), persimmon (5.8, 13.8), other oranges (3.9, 10.0), kiwi fruit (4.0, 8.5), pickled cucumber (1.6, 9.7), watermelon (2.0, 8.3), pears (1.8, 8.0), grapes (1.2, 8.4), strawberry (2.0, 7.0), pickled plum (1.7, 7.0), pickled Chinese cabbage (3.3, 5.0), peach (1.2, 5.6), pickled radish (2.2, 3.6), melon (0.9, 4.0), pickled eggplant (0.9, 2.8), pineapple (1.3, 2.0), pickled green vegetables (1.0, 0.7)		
2	egg (38.5, 37.5), pork (18.5, 30.8), chicken (20.9, 19.3), salad dressings (15.3, 22.3), cheeses (9.5, 26.0), bread (12.7, 22.4), mayonnaise (10.2, 14.4), milk (8.3, 11.9), ketchup (6.3, 8.8), jam (7.7, 4.3), Worcester sauce (5.2, 6.3), sausage (6.5, 4.6), bacon (3.0, 5.2), butter (2.0, 5.4), beef (2.7, 4.4), ham (1.1, 2.6), margarine (1.3, 2.4), pasta (1.4, 1.7)	wasabi (5.5, 8.0), salted fish (1.3, 7.7), mustard (4.0, 4.5), Japanese noodles (soba) (3.4, 4.5), Japanese noodles (udon) (2.3, 4.1), dried fish (0.7, 3.8), shrimp (1.2, 1.9), cod roe, salmon roe (0.7, 2.3), Chinese noodles (1.8, 1.1), Japanese noodles (soumen) (0.4, 1.9)			
3	onion (38.6, 46.8), carrot (24.6, 36.7), potatoes (18.3, 21.7), canned tuna (1.0, 2.9)	cabbage (28.8, 33.8), bean curd (19.8, 32.9), radish (23.0, 29.3), long green onion (21.8, 28.2), Chinese cabbage (17.9, 24.9), green pepper (16.6, 24.7), bean sprout (18.6, 17.9), seaweed (wakame) (13.7, 21.9), mushroom (shimeji) (13.0, 20.8), eggplant (13.4, 20.0), pumpkin (13.1, 17.7), mushroom (shitake) (13.7, 16.6), spinach (10.6, 16.5), mushroom (enoki) (10.3, 16.0) garlic (9.1, 14.9), deep-fried bean curd (9.1, 14.7), dried seaweed (nori) (7.7, 16.0), green vegetable (komatsuna) (8.4, 14.9), burdock (9.9, 12.8), sesame (3.7, 14.9), konjac (5.9, 10.5), green chive (7.6, 8.3), leek (5.8, 7.1), sweet potatoes (3.7, 6.0), seaweed (hijiki) (3.8, 4.3), fried bean curd (2.9, 4.1), yams (0.5, 5.4), green vegetable (shungiku) (1.1, 3.1), bean curd (koya-tofu) (0.6, 3.0), pollack, flatfish (0.4, 1.7), tunas, bonito (0.4, 1.1), horse mackerel, sardine (0.2, 0.6)	cucumber (19.5, 33.7), lettuce (20.4, 27.1), broccoli (17.6, 28.4), green asparagus (10.6, 19.7), snap bean (4.3, 10.4), pickled turnip (1.9, 3.7)		
4		amberjack (0.0, 0.2)			
5		rice cake (0.0, 0.4)			
6		taros (0.0, 0.3)			
7				sea breams (0.0, 0.0)	
8					eel (0.0, 0.0)

Note: Grouping of food items for male (rows) and female (columns) subjects. The row numbers and columns numbers are the sequential numbers of grouping in no particular order. The values in parenthesis are the sum of degree of simultaneous intakes $\sum \hat{p_{3}}n_{0}$ for male and female subjects in order.

**Table 4:** Ordered logit regression.

(**a**) **Female**												
	**X1 (Energy)**			**X2 (Carb)**			**X3 (Protein)**			**X4 (Lipid)**		
	**1**	**2**	**3**	**1**	**2**	**3**	**1**	**2**	**3**	**1**	**2**	**3**
[ Vigor ]												
Very active	-			-								
Full of energy											+	
Lively	-		+			+				-	+	
[ Anger/irritability ]												
Angry			-			-						
Inwardly annoyed or aggravated												
Irritable	++			++								
[ Fatigue ]												
Extremely tired			--			--			-			
Exhausted			-									
Weary or listless												
[ Anxiety ]												
Tense												
Worried or insecure	++			+			+					
Restless		++	--		++			+++	-			--
[ Depression ]												
Depressed												
Doing anything was a hassle												
Unable to concentrate							+	+		+		
Gloomy	++						+			+++		
Unable to handle work												
Sad										+		
[ Physical stress reaction ]												
Dizzy												
Joint pains									+			
Headaches												
A stiff neck and/or shoulders												
Lower back pain												
Eyestrain												
Heart palpitations or shortness of breath										+		
Stomach and/or intestine problems					--					+		
Lost my appetite	-			-								
Diarrhea and/or constipation		--			-					++		
Unable to sleep well								++			++	
(**b**) **male**												
	**X1 (energy)**			**X2 (carb)**			**X3 (protein)**			**X4 (lipid)**		
	**1**	**2**	**3**	**1**	**2**	**3**	**1**	**2**	**3**	**1**	**2**	**3**
[ Vigor ]												
Very active			+			+						
Full of energy							+					
Lively												
[ Anger/irritability ]												
Angry			--	+	+	-			--			--
Inwardly annoyed or aggravated					+							-
Irritable		+		+	+++	-						-
[ Fatigue ]												
Extremely tired										++		
Exhausted										++	+	
Weary or listless										++		
[ Anxiety ]												
Tense			-	-		-	-	--	-			
Worried or insecure												
Restless												
[ Depression ]												
Depressed			--		++	--						
Doing anything was a hassle			--		+	--				+		
Unable to concentrate												
Gloomy												
Unable to handle work												
Sad												
[ Physical stress reaction ]												
Dizzy										++	++	
Joint pains										++	+	
Headaches							-					
A stiff neck and/or shoulders								-		++		
Lower back pain										+	+	
Eyestrain				-			-					
Heart palpitations or shortness of breath					+					+		
Stomach and/or intestine problems	--			--			--				+++	
Lost my appetite										+		
Diarrhea and/or constipation	---			--			---	--				
Unable to sleep well	-			-			--		-			

Note: Signs and significances of estimated coefficients of ordered logit model regressing mental health on dependences on each food group. The plus and the minus signs indicates the positive and the negative association respectively. The number of signs denotes the significance of coefficients: one for 10%, two for 5%, and three for 1%. X1 (energy) through X4 (lipid) are substitute measures for dependence on each of 3 food groups shown in Table 3. X1 (energy), for example, is the rate of energy taken from each food group.

## Data Availability

The data obtained from the “*Sukoyaka* Health Survey” are available in a publicly accessible repository managed by the DNA Data Bank of Japan (DDBJ) Japanese Genotype-phenotype Archive at https://www.ddbj.nig.ac.jp/jga/index-e.html, accessed on 12 July 2024.

## Supplementary Materials

The following supporting information can be downloaded at https://www.mdpi.com/article/10.3390/nu16142296/s1: Table S1: Community detection in simultaneous food intake network; Table S2: Summary statistics of sample for ordered logit regression; Table S3: Results for confounders of ordered logit regression.

## Appendix A

**Table A1:** Transformation of stratified FFQ data.

Strata	FFQ	Monthly	Weekly
1	Less than 1 per month	0	0
2	1~2 per month	$1\leq x^{M}\leq 3$	
3	1~2 per week	$4\leq x^{M}\leq 8$	$1\leq x^{W}\leq 2$
4	3~4 per week	$9\leq x^{M}\leq 17$	$3\leq x^{W}\leq 4$
5	5~6 per week	$18\leq x^{M}\leq 26$	$5\leq x^{W}\leq 6$
6	1 per day	$27\leq x^{M}\leq 30$	$x^{W}=7$
7	2~3 per day	$31\leq x^{M}\leq 91$	$8\leq x^{W}\leq 22$
8	4~6 per day	$92\leq x^{M}$	$22\leq x^{W}$
9	7 or more per day		

Note: Transformation between monthly ($x^{M}$ ), weekly ($x^{W}$ ), and daily ($x^{D}$ ) numbers is based on the following formulas: $\begin{array}{cc} \left\lfloor \mathrm{upper bound of}\;x^{W}\times \frac{365.25}{7\times 12}\right\rfloor =\mathrm{upper bound of}\;x^{M}, & \left\lfloor \mathrm{upper bound of}\;x^{D}\times \frac{365.25}{12}\right\rfloor =\mathrm{upper bound of}\;x^{M},\\ \left\lfloor \mathrm{upper bound of}\;x^{M}\times \frac{12\times 7}{365.25}\right\rfloor =\mathrm{upper bound of}\;x^{W}, & \mathrm{upper bound of}\;x^{D}\times 7=\mathrm{upper bound of}\;x^{W}. \end{array}$

## Appendix B

In our model, the probability of $k_{i}$ times food i intake and $k_{j}$ times food j intake on $n_{0}$ occasions is

$$f\left(k_{i},\;k_{j}|p\right)\equiv \sum_{k=0}^{\min\left(k_{i},\;k_{j}\right)}\frac{n_{0}!p_{1}^{k_{i}-k}p_{2}^{k_{j}-k}p_{3}^{k}{\left(1-p_{1}-p_{2}-p_{3}\right)}^{n_{0}-k_{i}-k_{j}+k}}{\left(k_{i}-k\right)!\left(k_{j}-k\right)!\;k!\left(n_{0}-k_{i}-k_{j}+k\right)!}.$$

Observation $\left(X_{i},\;X_{j}\right),$ where $X_{i}$ and $X_{j}$ are the strata of the FFQ for food i and j, has the following log likelihood function:

$$l\left(X_{i},\;X_{j}|p\right)\equiv \left\{\begin{array}{c} \begin{array}{cc} \ln\sum_{k_{i}\in X_{i}}\sum_{k_{j}\in X_{j}}f\left(k_{i},\;k_{j}|p\right)+\ln\left(1-q_{i}\right)+\ln\left(1-q_{j}\right) & \left(X_{i},\;X_{j}\leq n_{0}\right) \end{array}\\ \begin{array}{cc} \ln\sum_{k_{j}\in X_{j}}\frac{n_{0}!p_{3}^{k_{j}}{\left(1-p_{3}\right)}^{n_{0}-k_{j}}}{k_{j}!\left(n_{0}-k_{j}\right)!}+\ln q_{i}+\ln\left(1-q_{j}\right) & \left(X_{i}>n_{0},\;X_{j}\leq n_{0}\right) \end{array}\\ \begin{array}{cc} \ln\sum_{k_{i}\in X_{i}}\frac{n_{0}!p_{3}^{k_{i}}{\left(1-p_{3}\right)}^{n_{0}-k_{i}}}{k_{i}!\left(n_{0}-k_{i}\right)!}+\ln\left(1-q_{i}\right)+\ln q_{j} & \left(X_{i}\leq n_{0},\;X_{j}>n_{0}\right) \end{array}\\ \begin{array}{cc} \ln q_{i}+\ln q_{j} & \left(X_{i},\;X_{j}>n_{0}\right) \end{array} \end{array}\right..$$

In the above equation, we use the symbols $X_{i}$ and $X_{j}$ to mean both the stratum of frequency observed in the data and the value of frequency unobserved in the data interchangeably.

Occasionally, the maximum likelihood estimate of $p_{3}$ turned out to be the corner solution $\hat{p_{3}}=0$, so the Hessian of the log likelihood is not invertible; hence, $\hat{\mathrm{Var}\left(\hat{p}\right)}$ cannot be obtained. In such cases, we set the *p*-value of $H_{0}:\;p_{3}\leq \left(p_{1}+p_{3}\right)\left(p_{2}+p_{3}\right)$ equal to 1 as a trivial value.

## Appendix C

Hypothesis test for significant combination of two foods:

**Table:**

Estimated model			Independent model		
	0	1		0	1
0	$1-p_{1}-p_{2}-p_{3}$	$p_{2}$	0	$\left(1-\pi_{1}\right)\left(1-\pi_{2}\right)$	$\left(1-\pi_{1}\right)\pi_{2}$
1	$p_{1}$	$p_{3}$	1	$\pi_{1}\left(1-\pi_{2}\right)$	$\pi_{1}\pi_{2}$

The rows indicate the states of food i and the columns food j; 0 and 1 mean eat and not eat, respectively. The left table describes the probability distribution of the four-state Bernoulli trial for each eating occasion. As the special case of the left table, the model in which intakes of food i and j are independent is shown in the right table. The inclusion of the right model in the left model is represented by $\pi_{1}=p_{1}+p_{3}$ and $\pi_{2}=p_{2}+p_{3}$. Foods i and j are significantly combined if $H_{0}:\;p_{3}\leq \pi_{1}\pi_{2}$ is rejected.

## Appendix D

Consolidation of FFQ items:

In food frequency questionnaire (FFQ) data, participants are asked about their frequency of intake for 128 foods in the same format of multiple-choice questions. Among these 128 items, 17 items are duplicated in terms of foodstuffs but subdivided by cooking methods. We consolidated these 17 items into 4 items: beef (steak, broiled, stir-fried, stewed), pork (stir-fried, deep-fried, stewed, simmered, soup, liver), chicken (broiled, stir-fried, simmered, deep-fried, liver), and bean curd (miso soup, boiled tofu).

To sum up the intake frequencies of each subdivided item, we considered the totals of the lower (upper) limits of the stratum for subdivided items as the lower (upper) limits of the stratum for consolidated items. The lower and upper limits of the stratum are indicated in Table A1. An example is the following:

An example

Beef steak: less than 1 per month	→	$x^{M}=0$	→	$\mathrm{Beef}:\;6\leq x^{M}\leq 14$
Beef (broiled): 1~2 per month	→	$1\leq x^{M}\leq 3$		
Beef (stir-fried): 1~2 per week	→	$4\leq x^{M}\leq 8$		
Beef (stewed): 1~2 per month	→	$1\leq x^{M}\leq 3$		

## Appendix E

**Table A2:** List of food items.

Meats 1. beef 2. pork 3. chicken 4. ham 5. sausage 6. bacon Fishes and shellfishes 7. salted fish 8. dried fish 9. canned tuna 10. salmon, trout 11. tunas, bonito 12. amberjack 13. pollack, flatfish 14. sea breams 15. horse mackerel, sardine 16. pacific saury, mackerel 17. shirasuboshi 18. cod roe, salmon roe 19. eel 20. squid 21. octopus 22. shrimp 23. clam, corb shell 24. fish sausage (chikuwa) 25. boiled fish paste (kamaboko) 26. fried fish paste (satsuma-age) Eggs and dairy products 27. low-fat milk 28. milk 29. egg 30. cheeses 31. yogurt Pulses 32. bean curd 33. bean curd (koya-tofu) 34. fried bean curd 35. deep-fried bean curd 36. fermented soybeans	Fruits 37. mandarin 38. other oranges 39. apple 40. persimmon 41. strawberry 42. grapes 43. melon 44. watermelon 45. peach 46. pears 47. kiwi fruit 48. pineapple 49. banana Vegetables 50. pickled radish 51. pickled green vegetables 52. pickled plum 53. pickled Chinese cabbage 54. pickled cucumber 55. pickled eggplant 56. pickled turnip 57. carrot 58. spinach 59. pumpkin 60. cabbage 61. radish 62. green pepper 63. tomatoes 64. long green onion 65. leek 66. green chive 67. green vegetable (shungiku) 68. green vegetable (komatsuna) 69. broccoli 70. onion 71. cucumber 72. eggplant 73. Chinese cabbage 74. burdock 75. bean sprout 76. snap bean 77. lettuce 78. green asparagus 79. garlic	Cereals 80. bread 81. Japanese noodles (udon) 82. Japanese noodles (soba) 83. Chinese noodles 84. pasta 85. Japanese noodles (soumen) 86. rice cake Potatoes 87. sweet potatoes 88. potatoes 89. taros 90. yams 91. konjac Confectioneries 92. Japanese cake 93. cakes 94. biscuit, cookie 95. chocolate 96. ice cream 97. snacks 98. rice cracker Miscellaneous 99. sesame 100. peanuts 101. mushroom (shitake) 102. mushroom (enokitake) 103. mushroom (shimeji) 104. seaweed (wakame) 105. seaweed (hijiki) 106. dried seaweed (nori) 107. butter 108. margarine 109. jam 110. salad dressings 111. mayonnaise 112. Worcester sauce 113. ketchup 114. mustard 115. wasabi

## References

[1] Chen P.-J., Antonelli M. (2020). Conceptual Models of Food Choice: Influential factors related to foods, individual differences, and society. Foods.

[2] Van Dyke N., Murphy M., Drinkwater E.J. (2024). We know what we should be eating, but we don’t always do that. How and why people eat the way they do: A qualitative study with rural Australians. BMC Public Health.

[3] Mai R., Hoffmann S., Hoppert K., Schwarz P., Rohm H. (2015). The spirit is willing, but the flesh is weak: The moderating effect of implicit associations on healthy eating behaviors. Food Qual. Prefer..

[4] Kagami-Katsuyama H., Sato-Ueshima M., Satoh K., Tousen Y., Takimoto H., Maeda-Yamamoto M., Nishihira J. (2023). The relationship between mental and physical minor health complaints and the intake of dietary nutrients. Nutrients.

[5] Brandes U., Delling D., Gaertler M., Gorke R., Hoefer M., Nikoloski Z., Wagner D. (2008). On modularity clustering. IEEE Trans. Knowl. Data Eng..

[6] Shimomitsu T., Haratani T., Nakamura K., Kawakami N., Hayashi T., Hiro H., Kato M. (2000). The final development of the Brief Job Stress Questionnaire mainly used for assessment of the individuals. Ministry of Labor Sponsored Grant for the Prevention of Work-related Illness: The 1999 Report.

[7] Inoue A., Kawakami N., Shimomitsu T., Tsutsumi A., Haratani T., Yoshikawa T., Shimazu A., Odagiri Y. (2014). Development of a short questionnaire to measure an extended set of job demands, job resources, and positive health outcomes: The New Brief Job Stress Questionnaire. Ind. Health.

[8] Holder M.D. (2019). The Contribution of Food Consumption to Well-Being. Ann. Nutr. Metab..

[9] Nishi D., Suzuki Y., Nishida J., Mishima K., Yamanouchi Y. (2017). Personal lifestyle as a resource for work engagement. J. Occup. Health.

[10] Deaton A., Muellbauer J. (1980). An Almost Ideal Demand System. Am. Econ. Rev..

[11] Hoenink J.C., Waterlander W.E., Mackenbach J.D., Mhurchu C.N., Wilson N., Beulens J.W.J., Nghiem M. (2021). Impact of taxes on purchases of close substitute foods: Analysis of cross-price elasticities using data from a randomized experiment. Nutr. J..

[12] Tian Y., Lautz S., Wallis A.O.G., Lambiotte R. (2021). Extracting complements and substitutes from sales data: A network perspective. EPJ Data Sci..

