# Supplementary material for: Internal Structure of Dietary Habits as a Restriction on Healthy Eating Policy in Japan
Source: Nutrients. 2024 Jul 17;16(14):2296. doi: 10.3390/nu16142296 (PMC11279819; doi:10.3390/nu16142296)
Supplement: Supplementary file 1 [file nutrients-16-02296-s001.zip › nutrients-3089417-supplementary.pdf]

Article

Internal Structure of Dietary Habits as a Restriction on Healthy Eating Policy in Japan

Supplementary Materials:

Table S1. Community detection in foods simultaneous intake network

	food	community		female				male			
		female	male	all	intra	inter	intra / all	all	intra	inter	intra / all
1	beef	1	2	4.4	2.0	2.4	45.6%	2.7	0.1	2.5	4.9%
2	pork	1	2	30.8	12.4	18.4	40.3%	18.5	1.7	16.8	9.0%
3	chicken	1	2	19.3	8.9	10.4	46.2%	20.9	3.6	17.3	17.0%
4	ham	1	2	2.6	1.4	1.2	54.3%	1.1	0.1	1.0	10.5%
5	sausage	1	2	4.6	3.5	1.1	76.7%	6.5	0.1	6.4	0.9%
6	bacon	1	2	5.2	2.8	2.4	53.5%	3.0	0.0	3.0	0.0%
7	salted fish	2	2	7.7	4.0	3.7	52.3%	1.3	0.9	0.3	72.6%
8	dried fish	2	2	3.8	1.9	1.8	51.8%	0.7	0.2	0.5	29.7%
9	canned tuna	1	3	2.9	1.4	1.5	47.4%	1.0	0.1	0.9	7.9%
10	salmon, trout	2	1	4.4	2.5	1.9	56.0%	1.8	0.2	1.6	8.6%
11	tunas, bonito	2	3	1.1	0.7	0.4	60.3%	0.4	0.1	0.3	20.5%
12	amberjack	2	4	0.2	0.2	0.1	63.3%	0.0	0.0	0.0	-
13	pollack, flatfish	2	3	1.7	0.9	0.8	53.5%	0.4	0.1	0.4	14.0%
14	sea breams	4	5	0.0	0.0	0.0	-	0.0	0.0	0.0	-
15	horse mackerel, sardine	2	3	0.6	0.5	0.1	77.8%	0.2	0.0	0.2	0.0%
16	pacific saury, mackerel	2	1	4.8	2.8	2.1	57.3%	0.9	0.0	0.9	5.3%
17	shirasuboshi	2	1	2.0	1.3	0.7	64.1%	0.3	0.0	0.3	0.0%
18	cod roe, salmon roe	2	2	2.3	1.4	1.0	58.9%	0.7	0.6	0.1	80.3%
19	eel	5	6	0.0	0.0	0.0	-	0.0	0.0	0.0	-
20	squid	2	1	0.6	0.5	0.1	77.6%	0.1	0.0	0.1	0.0%
21	octopus	2	1	0.5	0.4	0.1	73.2%	0.1	0.0	0.1	11.5%
22	shrimp	2	2	1.9	1.1	0.8	59.0%	1.2	0.6	0.7	44.7%
23	clam, corb shell	2	1	1.9	1.2	0.7	62.2%	0.7	0.1	0.6	14.5%
24	fish sausage (chikuwa)	2	1	5.9	2.8	3.1	47.7%	3.4	0.6	2.8	18.2%
25	boiled fish paste (kamaboko)	2	1	2.1	1.2	0.9	57.6%	1.9	0.4	1.6	18.8%
26	fried fish paste (satsuma-age)	2	1	1.4	0.9	0.5	66.9%	1.7	0.2	1.5	11.8%
27	low fat milk	1	1	8.5	3.6	4.9	42.5%	8.5	3.6	4.9	42.2%
28	milk	1	2	11.9	6.3	5.5	53.5%	8.3	1.7	6.7	19.9%
29	egg	1	2	37.5	14.9	22.6	39.6%	38.5	7.9	30.6	20.5%
30	cheeses	1	2	26.0	10.2	15.8	39.1%	9.5	3.4	6.1	35.5%
31	yogurt	1	1	33.2	12.9	20.3	38.9%	29.0	7.7	21.3	26.7%
32	bean curd	2	3	32.9	14.4	18.6	43.7%	19.8	4.5	15.3	22.7%
33	bean curd (koya-tofu)	2	3	3.0	2.0	1.0	67.2%	0.6	0.1	0.5	17.2%
34	fried bean curd	2	3	4.1	2.8	1.3	68.6%	2.9	0.2	2.8	5.6%
35	deep-fried bean curd	2	3	14.7	7.5	7.2	50.8%	9.1	0.6	8.5	6.3%
36	fermented soybeans	2	1	30.0	13.0	17.1	43.2%	20.5	4.7	15.8	22.9%
37	mandarin	3	1	25.9	8.5	17.5	32.6%	10.5	4.2	6.3	39.9%
38	other oranges	3	1	10.0	4.2	5.8	41.9%	3.9	1.4	2.6	34.3%
39	apple	3	1	19.2	6.9	12.3	36.0%	17.1	8.8	8.3	51.7%
40	persimmon	3	1	13.8	5.4	8.4	38.9%	5.8	1.5	4.3	25.1%
41	strawberry	3	1	7.0	3.3	3.7	47.1%	2.0	0.8	1.3	38.1%
42	grapes	3	1	8.4	3.8	4.6	45.4%	1.2	0.0	1.2	0.0%
43	melon	3	1	4.0	2.1	2.0	51.0%	0.9	0.4	0.5	39.7%
44	watermelon	3	1	8.3	3.6	4.8	42.9%	2.0	0.6	1.3	31.8%
45	peach	3	1	5.6	2.7	2.8	49.4%	1.2	0.4	0.8	36.9%
46	pears	3	1	8.0	3.6	4.5	44.5%	1.8	0.6	1.1	35.9%
47	kiwi fruit	3	1	8.5	3.3	5.2	39.1%	4.0	1.0	3.0	24.2%
48	pineapple	3	1	2.0	1.0	1.0	50.0%	1.3	0.6	0.7	47.3%
49	banana	3	1	18.1	5.4	12.7	30.0%	17.6	8.3	9.3	47.1%
50	pickled radish	3	1	3.6	1.4	2.3	37.1%	2.2	0.3	1.9	12.2%
51	pickled green vegetables	3	1	0.7	0.3	0.4	42.7%	1.0	0.3	0.7	27.0%
52	pickled plum	3	1	7.0	2.7	4.3	38.5%	1.7	0.4	1.3	24.3%
53	pickled Chinese cabbage	3	1	5.0	2.1	2.9	42.6%	3.3	1.4	1.9	42.1%
54	pickled cucumber	3	1	9.7	4.0	5.8	40.8%	1.6	0.7	0.9	42.3%
55	pickled eggplant	3	1	2.8	1.5	1.4	51.8%	0.9	0.4	0.5	46.8%
56	pickled turnip	3	3	3.7	2.0	1.7	53.0%	1.9	1.4	0.6	71.2%
57	carrot	1	3	36.7	12.2	24.5	33.3%	24.6	4.6	20.0	18.8%
58	spinach	2	3	16.5	7.7	8.8	46.7%	10.6	1.3	9.3	11.9%
59	pumpkin	2	3	17.7	7.6	10.1	43.1%	13.1	2.0	11.0	15.6%
60	cabbage	2	3	33.8	14.5	19.2	43.0%	28.8	5.9	22.9	20.6%
61	radish	2	3	29.3	14.0	15.3	47.7%	23.0	3.2	19.7	14.0%
62	green pepper	2	3	24.7	10.7	14.0	43.4%	16.6	2.5	14.1	15.0%
63	tomatoes	3	1	44.2	12.6	31.6	28.6%	28.9	16.5	12.5	56.9%
64	long green onion	2	3	28.2	12.5	15.7	44.2%	21.8	3.9	18.0	17.8%
65	leek	2	3	7.1	4.0	3.1	56.2%	5.8	0.0	5.8	0.0%
66	green chive	2	3	8.3	4.8	3.5	58.2%	7.6	1.0	6.6	13.6%
67	green vegetable (shungiku)	2	3	3.1	2.1	1.0	67.2%	1.1	0.0	1.1	0.0%
68	green vegetable (komatsuna)	2	3	14.9	7.4	7.6	49.4%	8.4	0.4	7.9	4.9%
69	broccoli	3	3	28.4	7.5	20.9	26.5%	17.6	11.0	6.6	62.6%
70	onion	1	3	46.8	16.0	30.8	34.1%	38.6	8.5	30.0	22.1%
71	cucumber	3	3	33.7	9.2	24.5	27.4%	19.5	12.3	7.2	63.0%
72	eggplant	2	3	20.0	9.6	10.4	47.8%	13.4	1.7	11.7	12.5%
73	Chinese cabbage	2	3	24.9	11.9	13.0	47.9%	17.9	2.6	15.3	14.5%
74	burdock	2	3	12.8	6.7	6.2	52.0%	9.9	0.9	8.9	9.4%
75	bean sprout	2	3	17.9	8.7	9.3	48.3%	18.6	4.4	14.2	23.8%
76	snap bean	3	3	10.4	3.5	6.9	33.2%	4.3	3.8	0.5	89.3%
77	lettuce	3	3	27.1	7.7	19.4	28.4%	20.4	11.6	8.9	56.6%
78	green asparagus	3	3	19.7	6.3	13.3	32.2%	10.6	7.7	3.0	72.2%
79	garlic	2	3	14.9	7.4	7.5	49.9%	9.1	0.5	8.6	5.2%
80	bread	1	2	22.4	12.3	10.2	54.7%	12.7	3.9	8.8	30.6%
81	Japanese noodles (udon)	2	2	4.1	2.2	2.0	52.4%	2.3	1.4	0.9	61.1%
82	Japanese noodles (soba)	2	2	4.5	2.7	1.8	59.6%	3.4	2.3	1.1	67.5%
83	Chinese noodles	2	2	1.1	0.8	0.4	65.9%	1.8	1.4	0.4	76.9%
84	pasta	1	2	1.7	0.9	0.9	49.9%	1.4	0.2	1.1	17.6%
85	Japanese noodles (soumen)	2	2	1.9	1.1	0.9	54.8%	0.4	0.2	0.1	64.7%
86	rice cake	2	7	0.4	0.2	0.2	55.4%	0.0	0.0	0.0	-
87	sweet potatoes	2	3	6.0	2.9	3.1	48.7%	3.7	0.3	3.4	8.1%
88	potatoes	1	3	21.7	7.3	14.4	33.5%	18.3	2.6	15.7	14.4%
89	taros	2	8	0.3	0.2	0.1	54.9%	0.0	0.0	0.0	-
90	yams	2	3	5.4	3.0	2.5	54.9%	0.5	0.1	0.5	11.9%
91	konjac	2	3	10.5	5.9	4.6	56.2%	5.9	1.2	4.7	21.1%
92	Japanese cake	1	1	3.4	1.4	2.0	42.2%	1.9	0.8	1.1	41.9%
93	cakes	1	1	1.3	0.6	0.7	45.3%	0.3	0.2	0.1	62.5%
94	biscuit, cookie	1	1	7.5	4.5	3.0	60.2%	1.7	1.2	0.5	69.7%
95	chocolate	1	1	17.7	9.2	8.5	51.9%	8.2	3.5	4.6	43.4%
96	ice cream	1	1	3.2	1.9	1.3	59.1%	1.4	0.9	0.5	61.8%
97	snacks	1	1	2.9	2.4	0.5	81.9%	1.6	0.8	0.8	50.7%
98	rice cracker	1	1	8.2	3.5	4.6	43.5%	3.7	1.9	1.8	51.4%
99	sesame	2	3	14.9	7.2	7.7	48.2%	3.7	0.2	3.6	4.4%
100	peanuts	1	1	2.6	0.9	1.7	35.7%	5.5	2.1	3.4	38.7%
101	mushroom (shitake)	2	3	16.6	8.4	8.1	50.8%	13.7	2.4	11.3	17.7%
102	mushroom (enokitake)	2	3	16.0	8.4	7.6	52.5%	10.3	1.7	8.6	16.0%
103	mushroom (shimeji)	2	3	20.8	10.2	10.5	49.3%	13.0	2.1	10.8	16.4%
104	seaweed (wakame)	2	3	21.9	10.3	11.6	46.8%	13.7	1.5	12.3	10.6%
105	seaweed (hijiki)	2	3	4.3	2.9	1.4	66.8%	3.8	0.6	3.3	14.8%
106	dried seaweed (nori)	2	3	16.0	7.4	8.6	46.5%	7.7	0.9	6.8	11.3%
107	butter	1	2	5.4	2.8	2.6	52.0%	2.0	0.6	1.4	28.5%
108	margarine	1	2	2.4	1.7	0.7	71.5%	1.3	0.1	1.2	4.6%
109	jam	1	2	4.3	2.4	1.9	55.6%	7.7	2.3	5.3	30.6%
110	salad dressings	1	2	22.3	8.5	13.8	38.1%	15.3	4.9	10.3	32.3%
111	mayonnaise	1	2	14.4	5.7	8.6	39.8%	10.2	2.4	7.8	23.5%
112	Worcester sauce	1	2	6.3	3.4	2.9	54.0%	5.2	1.0	4.2	18.9%
113	ketchup	1	2	8.8	4.1	4.7	47.0%	6.3	0.6	5.8	8.7%
114	mustard	2	2	4.5	2.7	1.8	59.8%	4.0	2.0	2.0	49.4%
115	wasabi	2	2	8.0	4.3	3.7	53.3%	5.5	3.1	2.4	56.4%

Note: The values are intra- and inter-community linkage in terms of the degree of simultaneous intake $\widehat{p_3} n_0$.

Table S2. Summary statistics of sample for ordered logit regression.

Explained variables	Female (N=587)							Male (N=205)						
	mean	s.d.	min	p25	p50	p75	max	mean	s.d.	min	p25	p50	p75	max
Vigor														
I have been very active	1.34	0.83	0	1	1	2	3	1.44	0.87	0	1	1	2	3
I have been full of energy	1.39	0.86	0	1	1	2	3	1.44	0.95	0	1	1	2	3
I have been lively	1.31	0.87	0	1	1	2	3	1.40	0.94	0	1	1	2	3
Anger-irritability														
I have felt angry	0.94	0.78	0	0	1	1	3	0.88	0.78	0	0	1	1	3
I have been inwardly annoyed or aggravated	0.97	0.77	0	0	1	1	3	0.87	0.81	0	0	1	1	3
I have felt irritable	0.93	0.79	0	0	1	1	3	0.83	0.77	0	0	1	1	3
Fatigue														
I have felt extremely tired	1.20	0.84	0	1	1	2	3	1.01	0.94	0	0	1	2	3
I have felt exhausted	0.72	0.87	0	0	0	1	3	0.57	0.80	0	0	0	1	3
I have felt weary or listless	1.07	0.87	0	0	1	2	3	0.86	0.89	0	0	1	1	3
Anxiety														
I have felt tense	0.84	0.86	0	0	1	1	3	0.75	0.78	0	0	1	1	3
I have felt worried or insecure	0.82	0.86	0	0	1	1	3	0.74	0.77	0	0	1	1	3
I have felt restless	0.52	0.71	0	0	0	1	3	0.50	0.69	0	0	0	1	3
Depression														
I have been depressed	0.67	0.81	0	0	0	1	3	0.58	0.73	0	0	0	1	3
I have thought that doing anything was a hassle	0.85	0.84	0	0	1	1	3	0.60	0.80	0	0	0	1	3
I have been unable to concentrate	0.65	0.71	0	0	1	1	3	0.69	0.77	0	0	1	1	3
I have felt gloomy	0.75	0.78	0	0	1	1	3	0.78	0.82	0	0	1	1	3
I have been unable to handle work	0.28	0.54	0	0	0	0	3	0.31	0.59	0	0	0	1	3
I have felt sad	0.47	0.71	0	0	0	1	3	0.31	0.54	0	0	0	1	3
Physical stress reaction														
I have felt dizzy	0.28	0.54	0	0	0	0	3	0.17	0.47	0	0	0	0	3
I have experienced joint pains	0.43	0.72	0	0	0	1	3	0.37	0.73	0	0	0	1	3
I have experienced headaches	0.67	0.75	0	0	1	1	3	0.40	0.66	0	0	0	1	3
I have had a stiff neck and/or shoulders	1.57	1.02	0	1	2	2	3	1.04	1.01	0	0	1	2	3
I have had lower back pain	0.93	0.94	0	0	1	1.5	3	0.71	0.86	0	0	0	1	3
I have had eyestrain	1.47	0.98	0	1	1	2	3	1.18	0.89	0	1	1	2	3
I have experienced heart palpitations or shortness of breath	0.26	0.54	0	0	0	0	3	0.19	0.48	0	0	0	0	3
I have experienced stomach and/or intestine problems	0.56	0.74	0	0	0	1	3	0.48	0.77	0	0	0	1	3
I have lost my appetite	0.16	0.43	0	0	0	0	2	0.17	0.46	0	0	0	0	2
I have experienced diarrhea and/or constipation	0.60	0.87	0	0	0	1	3	0.41	0.73	0	0	0	1	3
I haven't been able to sleep well	0.50	0.82	0	0	0	1	3	0.42	0.71	0	0	0	1	3

Explanatory variables	Female (N=587)					Male (N=205)				
	mean	s.d.	min	p50	max	mean	s.d.	min	p50	max
Energy1	0.39	0.11	0.12	0.39	0.78	0.17	0.10	0.02	0.15	0.52
Energy2	0.14	0.07	0.02	0.13	0.50	0.30	0.10	0.08	0.29	0.83
Energy3	0.03	0.03	0.00	0.02	0.19	0.04	0.02	0.00	0.04	0.14
Carbonhydrate1	0.32	0.15	0.07	0.30	1.02	0.19	0.13	0.01	0.16	0.83
Carbonhydrate2	0.14	0.08	0.02	0.13	0.59	0.23	0.12	0.01	0.21	0.90
Carbonhydrate3	0.07	0.06	0.00	0.05	0.44	0.06	0.04	0.00	0.05	0.22
Protein1	0.53	0.13	0.14	0.52	0.93	0.24	0.12	0.05	0.22	0.71
Protein2	0.28	0.11	0.04	0.27	0.73	0.48	0.13	0.14	0.48	0.86
Protein3	0.02	0.02	0.00	0.02	0.16	0.09	0.05	0.00	0.08	0.31
Lipid1	0.55	0.12	0.11	0.55	0.95	0.19	0.11	0.02	0.18	0.68
Lipid2	0.13	0.08	0.00	0.12	0.67	0.45	0.12	0.15	0.45	0.78
Lipid3	0.00	0.00	0.00	0.00	0.02	0.04	0.03	0.00	0.03	0.15

Table S2. (continued)

Confounders										
	Female (N=587)					Male (N=205)				
	mean	s.d.	min	p50	max	mean	s.d.	min	p50	max
age	50.1	11.3	21.0	51.0	76.0	53.4	12.6	21.0	54.0	76.0
BMI	21.5	3.3	15.0	20.8	38.6	23.6	3.5	16.2	23.2	43.8
SBP	114.4	16.6	77.0	112.0	199.0	127.9	17.7	94.0	127.0	190.0
Housemate										
Spouse	0.56	0.50	0	1	1	0.83	0.37	0	1	1
Child	0.41	0.49	0	0	1	0.42	0.49	0	0	1
Parent	0.16	0.36	0	0	1	0.06	0.24	0	0	1
Living alone	0.20	0.40	0	0	1	0.11	0.31	0	0	1
# of housemates	1.54	1.18	0	1	6	1.65	1.12	0	1	6
Caregiving	0.07	0.26	0	0	1	0.03	0.17	0	0	1
School										
Two-year college	0.49	0.50	0	0	1	0.15	0.36	0	0	1
University	0.25	0.43	0	0	1	0.66	0.48	0	1	1
Occupation										
Manager	0.01	0.10	0	0	1	0.16	0.36	0	0	1
Specialist	0.21	0.41	0	0	1	0.24	0.43	0	0	1
Clerical	0.21	0.41	0	0	1	0.18	0.38	0	0	1
Employment										
Full-time	0.27	0.44	0	0	1	0.54	0.50	0	1	1
Part-time	0.36	0.48	0	0	1	0.19	0.39	0	0	1
Health checkup	0.80	0.41	0	1	2	0.89	0.32	0	1	1
Dental checkup	0.60	0.50	0	1	2	0.52	0.51	0	1	2

Note: For explained variables, values are: 0=" Almost never", 1="Sometimes", 2="Often", and 3="Almost always". For explanatory variables, see text for details. As for confounders, SBP is systolic blood pressure.

Table S3. Results for confounders of ordered logit regression

Female	age	BMI	SBP	Housemate					Caregiving	School			Occupation		Employment		Health checkup	Dental checkup
				Spouse	Child	Parent	Living alone	# of housemates		Two-year college	University	Manager	Specialist	Clerical	Full-time	Part-time		
Vigor																		
I have been very active				++					−	++	+							
I have been full of energy			+		+													
I have been lively									−									
Anger-irritability																		
I have felt angry	−−−									−	−−	++						−
I have been inwardly annoyed or aggravated	−−−	+		+					+++	−		+++						
I have felt irritable	−−−	++		+					+				+					
Fatigue																		
I have felt extremely tired	−−−								++								++	−−
I have felt exhausted	−−−																++	−−−
I have felt weary or listless	−−−												++					
Anxiety																		
I have felt tense	−						−−−		++	−							+	
I have felt worried or insecure	−−−								++									
I have felt restless		+																
Depression																		
I have been depressed	−−−	+								−−								
I have thought that doing anything was a hassle	−−										+							
I have been unable to concentrate																++		
I have felt gloomy			−−						+	−−−								
I have been unable to handle work	−	++												−−				
I have felt sad			−						++	−								
Physical stress reaction																		
I have felt dizzy	−−	+															+	
I have experienced joint pains	++	++															+	
I have experienced headaches	−−−	++				−							++					
I have had a stiff neck and/or shoulders		++													++			
I have had lower back pain		++										++						
I have had eyestrain		+												+++				
I have experienced heart palpitations or shortness of breath											−−			−				
I have experienced stomach and/or intestine problems																		
I have lost my appetite		−															−	
I have experienced diarrhea and/or constipation											−−−		+					
I haven't been able to sleep well		+			−−				++						−			

Table S3. (continued)

Male	age	BMI	SBP	Housemate					Caregiving	School		Occupation			Employment		Health checkup	Dental checkup
				Spouse	Child	Parent	Living alone	# of housemates		Two-year college	University	Manager	Specialist	Clerical	Full-time	Part-time		
Vigor																		
I have been very active		+		−								−		−−−			++	
I have been full of energy			++	−−−		−						−−		−−			+	
I have been lively				−−								−−		−−			+	
Anger-irritability																		
I have felt angry	−−									+++								
I have been inwardly annoyed or aggravated	−−−									++								
I have felt irritable	−−−			++						++								
Fatigue																		
I have felt extremely tired	−−−								++	++								
I have felt exhausted	−−−									+								
I have felt weary or listless	−−−													+				
Anxiety																		
I have felt tense	−−								+++							−		
I have felt worried or insecure	−−−	−−							+++	++								
I have felt restless	−−−		++						+++									
Depression																		
I have been depressed	−−−			+					++	+++								
I have thought that doing anything was a hassle	−−−			+++						+								
I have been unable to concentrate	−−−			+++			+		++									
I have felt gloomy	−−−			+					+	+								
I have been unable to handle work	−−−													++				
I have felt sad	−−−						−	−										
Physical stress reaction																		
I have felt dizzy																		
I have experienced joint pains	+																	
I have experienced headaches	−−	−												+				
I have had a stiff neck and/or shoulders	−								++									
I have had lower back pain					−			++	++			+						
I have had eyestrain												+	++	++				
I have experienced heart palpitations or shortness of breath									++									
I have experienced stomach and/or intestine problems						+		−		−−	−−−				++			
I have lost my appetite			−−			+					−−	+++	+++	++				
I have experienced diarrhea and/or constipation																		
I haven't been able to sleep well	−−														−−			

Note: The number of signs denotes the significance of coefficients. One for 10%, two for 5%, tree for 1%. The variables in columns are confounders in the ordered logit regressions in Table 4. The items of occupational stress test displayed in rows are dependent variables of the regressions.
